# Coverage of azithromycin mass treatment for trachoma elimination in Northwestern Ethiopia: a community based cross-sectional study

**DOI:** 10.1186/s12886-018-0868-1

**Published:** 2018-08-06

**Authors:** Zelalem Tilahun, Teferi Gedif Fenta

**Affiliations:** Social and Adminstrative Pharmacy Working Group, Departement of Pharmaceutics and Social Pharmacy, College of Health Sciences, Addis Ababa University, P.O.Box 1176, Addis Ababa, Ethiopia

**Keywords:** Azithromycin mass treatment, Coverage, Trachoma elimination, Northwest Ethiopia

## Abstract

**Background:**

Mass drug administration with antibiotics predominantly with azithromycin is one of the four arms of the SAFE strategy. The elimination of ocular chlamydial infection is only achieved as long as the azithromycin mass treatments (AMT) are given frequently enough and at a high enough coverage. This study was conducted to assess the coverage of azithromycin mass treatment and its determinants in Awi Zone, Northwestern Ethiopia.

**Methods:**

House to house survey using a structured questionnaire was done between July 7 to July 25, 2013. Coverage is defined as the proportion of individuals in the eligible population who actually ingested the Azithromycin during the Campaign.

**Results:**

A total of 1267 households were enrolled in the survey in which 5826 eligible members were living in these households. Almost half (54.6%) of the community members who were eligible for all six campaigns had participated in more than three campaigns of azithromycin mass treatment. The overall average self-reported coverage of the azithromycin mass treatment (AMT) in all six campaigns was 62.8% (64% in rural vs. 61.6% urban). On average, each eligible person had taken the drug 3.77 times. The rural residents were significantly more likely to have received treatment during the last round of AMT in 2012 {AOR = 2.35; 95% CI [1.80–3.06]}. Azithromycin uptake status of female household heads was less than the corresponding male household heads {AOR = 0.41; 95% CI [0.24–0.72]}. Household heads’ awareness about trachoma (AOR = 2.55; 95% CI [1.19–5.44]) and AMT {AOR = 7.19; 95% CI [3.27–15.82]} had positive association with acceptability.

**Conclusion:**

The overall average AMT coverage was found to be low. There was low coverage of the treatment in the urban community as compared to the rural residents. Misconceptions of household heads about trachoma and azithromycin have negatively affected the coverage. Further work on why female household heads are associated with higher risk of non-participation in AMT is warranted. Strengthening awareness creation and consideration of additional campaigns is essential.

**Electronic supplementary material:**

The online version of this article (10.1186/s12886-018-0868-1) contains supplementary material, which is available to authorized users.

## Background

“Trachoma, a neglected tropical disease, is the world’s leading infectious cause of blindness” [1]. Globally, 1.2 billion people live in trachoma endemic areas; of these, 48.5% of the global burden is concentrated in Ethiopia, India, Nigeria, Sudan and Guinea [2]. In Ethiopia, approximately 67 million people are at risk for trachoma [3]. The Amhara National Regional State (ANRS) of Ethiopia was the most trachoma-endemic regional states in Ethiopia. Awi zone is one of the trachoma endemic zones among the 10 zonal administrations in ANRS with a TF and TT prevalence of 38.9 and 5.4%, respectively [3, 4].

For many years, topical agents such as tetracycline were used as a treatment of choice for ocular infection with *C.trachomatis* because it is devoid of systemic side effects in children [5]. However, effective treatment requires that the drug be taken every day for four to six weeks [6]. Moreover because of its oily base, its use may be associated with blurred vision. Due to these reasons, compliance with these agents is poor [7].

Many randomized control trials indicated that azithromycin is the treatment of choice for ocular infection with *C.trachomatis* [2, 8–10]. Azithromycin is also associated with a short-term reduction in diarrheal morbidity in children [11, 12]. It is easy to administer and higher coverage may be possible as compared to tetracycline topical treatment [13].

The World Health Assembly passed a resolution to eliminate blinding trachoma by implementing the “SAFE” (S = surgery, A = antibiotic, F = facial cleanliness and E = environmental improvement) strategy [14]. In Ethiopia the first strategic plan for trachoma control was developed in 2005 after a pilot trial of the SAFE strategy in four districts of ANRS. Then, ANRS Health Bureau partnering with the Lions Club of Ethiopia and the Carter Center had expanded trachoma control efforts from the four pilot districts to all districts with the SAFE strategy [3].

Mass drug administration with antibiotics predominantly with azithromycin is one of the four arms of the SAFE strategy. WHO had recommended community-wide distribution of oral azithromycin when the prevalence of trachomatous inflammation follicular (TF) is greater than 10% in children aged 1**–**9 years, and trichiasis prevalence exceeding 1% in persons aged over 14 years [15]. Full participation is necessary for maximizing the impact of trachoma control programs [16]. Antibiotic distribution teams should offer azithromycin to all individuals over the age of six months in eligible communities. Overall coverage should be as high as possible, but treatment of 80% of the resident population should be the minimum target [17].

Unlike patient oriented treatments that are commonly self-initiated, there may not be full acceptance of the community for drugs that are given in the form of campaign due to different reasons [18, 19]. In Ethiopia, even if there are many studies addressing the effectiveness of azithromycin mass treatment (AMT) for trachoma control, its acceptability and determinants has not been well explored. This study was therefore conducted to assess the acceptability of the azithromycin mass treatment and its determinants in Awi zone, Northwestern Ethiopia.

## Methods

### Study area and design

A community based cross sectional survey was conducted following 6 AMT rounds in both urban and rural *kebeles* (the smallest administrative units in the government structure) of Injibara town and Adjacent Banja districts from July 7 to July 25, 2013.

### Study participants

All individuals living in Injibara town and Banja districts were taken as source population*.* The study population was all individuals who live in the selected two urban and six rural kebeles. All family members who lived more than 8 months in that household during the time of the survey were included. All children who were older than 6 months during 2012 AMT program were also taken as study participants.

### Sample size determination

The number of households to be involved in the survey was determined using the single proportion formula [20]. The sample size was calculated with the assumption that the level of coverage was 0.5 and the absolute sampling error to be tolerated as 0.04 with 95% confidence interval. Taking the experiences of similar African Studies the design effect of 2 was considered to calculate the sample size [21]. Adding 10% for non-responses, a total of 1321 households were included.

### Sampling method

The number of households covered in urban and rural *kebeles* were allocated based on proportionate to the size of population established in 2007 CSA census (28% Urban and 72% Rural) [22]. Accordingly, six rural and two urban *kebeles* were randomly selected. Finally, the number of households in each *kebele* was determined proportionate to the size of households in each *kebele* and specific households were selected using a systematic random sampling technique.

### Operational definition

#### Coverage

Coverage is defined as the proportion of individuals in the sampled population who actually ingested the Azithromycin during the Campaign.

#### Azithromycin mass treatment (AMT)

It is an annual mass administration of azithromycin to all eligible community members (children less than 6 months old are excluded) for the purpose of trachoma elimination. Six rounds of mass distribution were conducted by Health Extension Workers in the Districts and the last campaign was in November 2012.

#### Serious adverse effects

An adverse experience following AMT that results in death, life threatening condition, in-patient hospitalization, disability and/or birth defects.

#### Data collection instruments

Adult household members were interviewed with a pretested structured questionnaire (attached as Additional file 1) by 13 trained data collectors. The structured questionnaire was translated to Amharic (official language) and most of the interview was done using Amharic. For those participants who couldn’t speak and understand Amharic, interview was done using the local language. The head of each household was interviewed about himself and eligible children less than 18 years old as well as for family members who were absent during the data collection time. Data collectors with health background were carefully selected and given training on how to administer the questionnaire to avoid possible interviewer bias.

#### Data analysis and interpretation

Data was coded and entered into Epi Info version 3.5.3 by three trained data entry clerks. Then, the data was transferred to SPSS version 20.0 and analyzed. Socio-demographic variables including sex, age, marital status, educational status, occupation and place of residence, and awareness about AMT were considered as independent variables and frequency of self reported AMT coverage as outcome variable. In addition to simple descriptive statistics, chi square test, correlation and logistic regression analysis were conducted to show possible associations. *P*-value< 0.05 was considered as statistically significant.

## Results

A total of 1321 households were enrolled in the survey. Complete questionnaires were obtained from1267 households; making a response rate of 96%. All members in the 1267 households who fulfilled the inclusion criteria were included and hence a total of 5826 eligible household members were considered in the subsequent analysis. Among these, 5266 (90.4%) were greater than or equal to 7 years old at the time of the survey and anticipated as eligible for all six rounds.

### Socio-demographic characteristics

Among the total 1267 households included in the study, 897 (70.8%) of them were from rural areas. Most of the household heads 973 (76.8%) were males. The mean age of household heads was 46.3 ± 14.2. The mean age of the AMT eligible participants was 25.1 ± 17.64. More than half of the participants were less than 20 years (Table 1).

**Table 1:** Socio demographic distribution of the study participants, Injibara town and Banja districts of Awi zone, Northwestern Ethiopia, July 2013

Socio demographic variables *N* = 5826	Urban residents	Rural residents	Total *n* (%)
1. Sex			
Male	706	2172	2878 (49.4)
Female	804	2144	2948 (50.6)
2. Age			
1–5 years	135	318	453 (7.8)
6–10 years	208	608	813 (14)
11–15 years	207	727	934 (16)
16–20 years	224	696	920 (15.8)
21–40 years	537	1066	1603 (27.5)
41–60 years	169	671	840 (14.4)
> 60 years	30	233	263 (4.5)
3. Occupation			
Student	635	1679	2314 (39.7)
Farmer	51	1791	1842 (31.6)
Merchant	100	45	145 (2.5)
Government employee	245	36	281 (4.8)
House wife	182	181	363 (6.2)
Jobless	56	32	88 (1.5)
Others^a^	241	552	793 (13.6)
4. Religion			
Orthodox	1497	4276	5773 (99.1)
Muslim	0	8	8 (0.13)
Protestant	13	1	14 (0.24)
Others^b^	0	31	31 (0.53)
5. Educational Status			
Unable to read and write^c^	336	1970	2306 (39.6)
Able to read and write	97	322	419 (7.2)
Grade 1–4	167	604	771 (13.2)
Grade 4–8	356	964	1320 (22.6)
Grade 9–12	261	412	673 (11.6)
College and Above	293	44	337 (5. 8)
6. Marital Status			
Single^d^	871	2562	3433 (58.9)
Married	573	1562	2135 (36.7)
Divorced	32	86	118 (2.0)
Widowed	34	106	140 (2.4)

^a^Children < 5 years, daily laborer, handicraft, local beer maker, monk, tailor

^b^Catholic, paganism

^c^ (This category includes preschool children)

^d^ (This category includes children)

### Household heads’ awareness and perception about trachoma

Among the total 1267 household heads, 1203 (94.9%) had ever heard about trachoma. The main information source for most of the household heads about trachoma was health professionals 1076 (89.44%) followed by neighbors 87 (7.23%). Poor environmental sanitation was mentioned by 717 (59.6%) of them as a cause of trachoma. Among 1203 household heads who had ever heard about trachoma, 428 (35.6%) mentioned fly as a way of transmission; 401 (33.3%) of them did not know the way of trachoma transmission whereas 22 (1.8%) household heads believed that trachoma is a non-communicable disease. But a sizable proportion 1084 (90%) believed that trachoma is a preventable disease. However, 77 (7.1%) of them did not know any prevention methods for trachoma.

### Household heads’ awareness and perception about AMT

From the total household heads participated in the survey, 1234 (97.4%) of them had ever heard about AMT administrated for the elimination of trachoma. Among these, 1117 (90%) had heard about it from health professionals. Almost all of them 1263 (99.7%) reported that mass drug treatment had ever been given in their *kebeles* even though some of them were not aware about the treatment and its purpose. Of the total respondents, 150 (11.8%) believed that the drug was not given based on free will. Among the total household heads participated in the study, 1204 (95%) reported that they would volunteer to participate in the mass treatment if it was to be continued in the future. The most frequently mentioned reasons to end their participation to AMT by the rest of 63 (5%) household heads were fear of illness and death by the drug, serious side effect in previous campaign, having chronic illness, and absence of change to their eye health.

### Self-reported azithromycin mass treatment coverage

In 1240 (97.9%) of the households, it was reported that at least one member had ever participated in any rounds of the campaign and the rest 27 (2.1%) claimed that none of the members had ever taken AMT. Being absent from home during the campaigns, health problems, and fear of the severe side effects were the major reasons mentioned for non-participation in AMT.

**Fig. 1:**
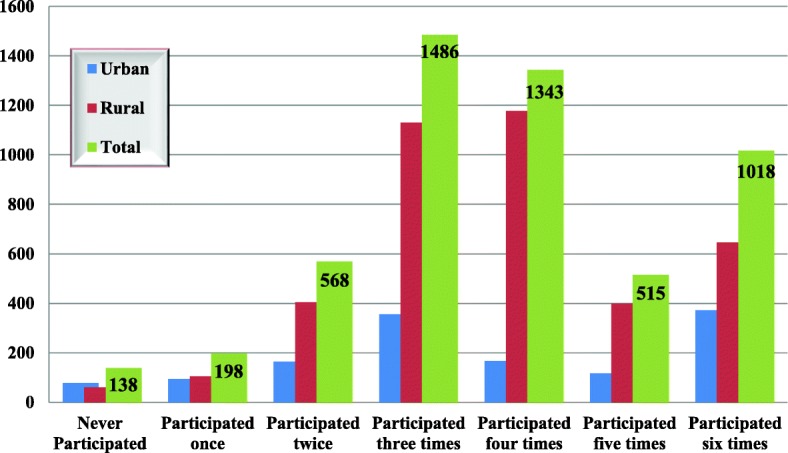
Frequency of AMT self-reported uptake among urban and rural household members in Injibara town and Banja districts of Awi Zone, July 2013

From the total 5266 (90.4%) household members who were eligible for all campaigns, only 2876 (54.6%) of them claimed to have participated more than three times out of the six rounds of AMT campaigns. The overall average self-reported coverage of the AMT in all six campaigns was 62.8% (64% rural and 61.6% urban). On average, each eligible person participated 3.77 times. From the total 560 children who were not eligible for all campaigns, 75 children were less than 1 year old and expected to participate in the campaign once. Of these, only 54 (72%) were taken the treatment. From 1510 AMT urban household members enrolled in the study, 1344 (89%) were eligible for all campaigns. Of these 78 (5.8%) of them had never participated and only 72 (27.7% completed the six rounds of AMT (Fig. 1).

The self-reported AMT coverage of the 2012 campaign was 92.9%; whereas a report obtained from District Health Office indicated that the coverage was close to 96%.

Out of the total 1227 household heads who had ever taken azithromycin, 1166 (95%) believed that AMT was beneficial. Among those who said AMT is beneficial, 978 (83.9%) related the benefit to eye health; 481 (41.3%) for eradicating intestinal worm; and 15 (1.3%) for improving the general health. Other benefits such as treatment for hemorrhoid and stimulating appetites were mentioned by 5 (0.4%) of the respondents.

### Reported side effects with AMT use

**Fig. 2:**
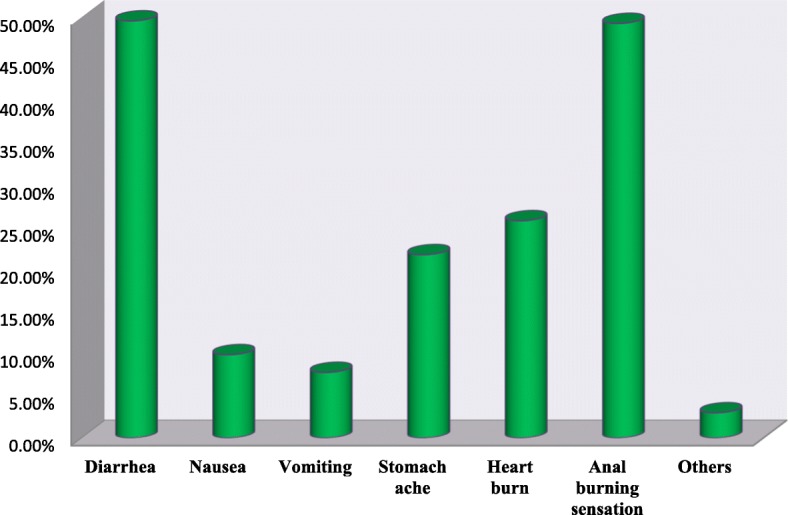
Types of side effects reported by household heads after they took AMT in Injibara town and Banja districts of Awi zone, July 2013 (*N* = 538)

From the total 1227 household heads who had ever taken the drug, 538 (43.9%) reported to have had side effects of azithromycin. Anal burning sensation, diarrhea, and heartburn were the three most prevalent side effects reported (Fig. 2).

Out of the total 538 household heads who had ever encountered side effects of azithromycin, 83 (15.4%) had claimed that they experienced with serious adverse effects of the drug. In addition, 296 (23.4%) of the household heads had heard other persons complaining about the drug’s side effects. Of which, 88 (29.7%) of them reported that they had ever heard about serious adverse effects of the drug such as birth defect, inpatient hospitalization and disability. From this, 38 (43.2%) of them reported that they knew persons who encountered with at least one of the serious adverse effects of the drug.

From the total 1227 household heads who had ever participated in AMT, 66 (5.4%) of them used the drug for different purposes other than trachoma such as for treating intestinal worms, promote animal fertilization and for the relief of abdominal pain.

### Determinants of the 2012 azithromycin mass treatment coverage

The 2012 Azithromycin uptake was not found to be influenced by socio demographic characteristics, except job. Students’ participation in the mass treatment was higher as compared to farmers, merchants and others. Place of residence affects the drug uptake status. In this regard, the proportion of eligible rural residents who took AMT more than three times was 56.7% and for urban residents, it was 48.7%. This difference was statistically significant {AOR = 2.35, 95% CI [1.80–3.06]}.

There was a significant difference between the rural residents and urban residents in the number of times they took azithromycin. Higher number of rural residents had taken azithromycin more than 3 times as compared to urban residents {AOR = 1.69; 95% CI [1.44–1.98]}. There was no significant difference in frequency of AMT uptake between males and females. There was a significant difference in the frequency of AMT uptake among children ages between 7 to 10 years compared to other age groups. In this regard, the proportion of adults with age between 16 and 20 years who took azithromycin more than 3 times was higher than in children with age between 7 to 10 years {AOR = 2.77; 95% CI [2.15–3.57]}. Similarly, the study participants who were older than 20 years were at better enactment in taking azithromycin more than 3 times as compared to children less than 15 years. There was also a positive correlation between the number of times they took azithromycin and age (*r* = 0.235; 95%CI, *p* = 0.01). As age increased, participation in AMT increased but being literate had no effect in participation rate (Table 2).

**Table 2:** Socio demographic factors associated with the frequency of AMT uptake among eligible participants to all campaigns, in Injibara town and Banja districts of Awi zone, July 2013

Socio demographic variables [*N* = 5266]	Frequency of AMT uptake		95% Confidence Interval	
	> 3 Times	≤ 3 Times	COR	AOR
Residence				
Rural	2222	1700	1.37 [1.22–1.56]	1.69 [1.44–1.98]
Urban	654	690	1.00	
Sex				
Female	1450	1217	0.98 [0.88–1.09]	0.92 [0.82–1.04]
Male	1426	1173	1.00	
Age				
> 60 years	165	98	3.70 [2.75–4.97]	4.40 [2.96–6.56]
41–60 years	532	309	3.78 [3.06–4.67]	4.71 [3.38–6.58]
21–40 years	968	634	3.35 [2.78–4.04]	4.04 [3.01–5.42]
16–20 years	543	377	3.16 [2.47–3.89]	2.77 [2.15–3.57]
11–15 years	447	487	2.01 [1.64–2.47]	1.76 [1.40–2.22]
7–10 years	221	485	1.00	1.00
Job				
Farmer	1074	734	1.45 [1.28–1.64]	1.04 [0.81–1.33]
Merchant	83	62	1.32 [0.94–1.86]	1.03 [0.70–1.52]
Government employee	156	125	1.23 [0.96–1.58]	0.94 [0.62–1.43]
Housewife	234	129	1.79 [1.42–2.26]	1.49 [1.08–2.06]
Jobless	48	40	1.19 [0.77–1.82]	1.00 [0.63–1.58]
Others	140	173	0.80 [0.630.28–1.04]	0.77 [0.58–1.03]
Student	1141	1127	1.00	1.00
Educational Status				
College and above	183	154	0.87 [0.69–1.10]	1.26 [0.85–1.85]
Grade 9–12	416	257	1.19 [0.99–1.43]	1.78 [1.39–2.28]
Grade 4–8	712	607	0.86 [0.75–0.99]	1.21 [0.98–1.50]
Grade 1–4	288	449	0.47 [0.40–0.56]	0.62 [0.49–0.78]
Able to read and write	240	162	1.09 [0.87–1.36]	1.01 [0.79–1.28]
Unable to read and write	1037	761	1.00	1.00
Marital Status				
Married	1277	853	1.51 [1.35–1.69]	1.41 [1.15–1.74]
Divorced	79	39	2.05 [1.38–3.02]	1.89 [1.21–2.94]
Widowed	88	52	1.71 [1.20–2.43]	1.54 [1.02–2.34]
Single	1432	1446	1.00	1.00

### Factors associated with participation in the 2012AMT

The self-reported participation rate of rural household heads in the 2012 mass treatment was higher than their urban counterparts (AOR = 2.33; 95% CI [1.07–5.10]). Azithromycin uptake status of female household heads was significantly less than the corresponding male household heads {AOR = 0.41 95% CI [0.24–0.72]}. Occupation, educational status and marital status of household heads did not affect their uptake status. However, their participation was influenced by their awareness and perception about AMT. For example, those household heads who had ever heard about AMT were more likely participated in the 2012 campaign as compared to those who were unaware {AOR = 7.19;95% CI [3.27–15.82]}. In addition, the proportion of household heads who believed that AMT was given on free will and had participated in 2012 mass campaign was more than the proportion of those who considered AMT as an obligation {AOR = 2.93; 95% CI [1.77–4.86]} (Table 3).

**Table 3:** Association between awareness about AMT and self-reported uptake status of household heads in 2012, Awi Zone, July 2013

Variables	Took 2012 AMT		95% Confidence Interval	
	Yes	No	COR	AOR
Heard about AMT				
Yes	1125	109	10.97 [5.39–22.32]	7.19 [3.27–15.82]
No	16	17	1.00	
Did they Know why AMT given in free?				
Yes	764	79	1.21 [0.82–1.77]	0.97 [0.63–1.48]
No	377	47	1.00	1.00
Was the treatment given to those willing to take?				
Yes	1024	93	3.11 [2.0–4.83]	2.93 [1.77–4.86]
No	117	33	1.00	1.00
Did they think that the drug is beneficial for trachoma?				
Yes	1100	87	12.03 [7.37–19.63]	7.33 [4.13–13.02]
No	41	39	1.00	1.00
Did they experience any side effects’ of the drug?				
Yes	495	43	1.00	1.00
No	646	43	1.31 [0.84–2.02]	1.40 [0.87–2.26]
Had they got SAE of the drug?				
Yes	75	8	0.78 [0.35–1.75]	0.87 [0.35–2.12]
No	419	35	1.00	1.00
Heard drug’s side effects from other Persons?				
Yes	257	39	1.00	1.00
No	884	87	1.54 [1.03–2.31]	1.34 [0.78–2.32]
Did they know a person with SAE of the drug?				
Yes	32	6	1.02 [0.32–3.22]	1.16 [0.24–5.57]
No	42	8	1.00	
Did treatment providers provide any information?				
Yes	852	40	3.39 [2.17–5.29]	2.49 [1.53–4.08]
No	289	46	1.00	
Willingness to take the drug in the future				
Yes	1113	91	15.29 [8.90–26.26]	5.78 [2.44–13.68]
No	28	35	1.00	

*SAE* Serious adverse effect

## Discussion

The 2012 self reported azithromycin mass treatment coverage was 92.9%. This was higher than 80%, the minimum attainable coverage set by WHO [17]. This finding was also higher than 76% taken from the mass treatment coverage in Tanzania [19]; and the mean treatment coverage in 48 eligible communities in four Gambian districts taken at baseline, and after one and two years [21].

The overall self-reported coverage of AMT for the six campaigns in the study area was estimated based on the number of times that each eligible household member had taken the treatment. In this regard, the proportion of eligible household members for all campaigns who took azithromycin more than three times out of the six rounds was 54.6%. The reported mean number of times that each person had taken azithromycin was 3.77 ± 1.51. Even though the self-reported coverage might be liable to recall bias, the 2012 AMT data obtained from the official records of the district health office was higher than the self-reported coverage for the same year (96% vs. 92.8%). A similar type of discrepancy was observed in a study conducted in Plateau State of Nigeria, in which only 60.3% of the participants reported to have received azithromycin or tetracycline eye ointment during mass drug administration but the coverage report taken from administrative data was 75.8% [23]. The reasons for such differences should be explored in the future.

There was a report from the community that the drug manifested side effects. Consequently, even all the community members who had received the drug might have not taken it; resulting a decrease in the overall actual coverage below the minimum WHO target [17]. This may have negative impact in the program success as intended elimination can only occur if the mass treatment is given frequently enough and at a high coverage. This is due to the fact that treatment of few persons in endemic areas may result in reinfection from family or neighborhood sources unless the treatment is more widespread [24–26]. Therefore, the present study finding is an indicative of the need for determining the prevalence of TF and consider additional campaigns of AMT in the studied communities.

This study also showed that the proportion of eligible rural residents who took azithromycin more than three times in the six campaigns was higher as compared to urban residents (AOR = 1.69; 95% CI [1.44–1.98]). Better acceptance and coverage of the program in the rural community is more appreciable since the prevalence of trachoma in the rural population showed a fourth fold increase as compared to the urban [4].

Older age groups were more likely to participate in AMT than the younger age group. A bivariate correlation analysis also showed that there is positive correlation between the number of times they participated in AMT campaigns and age. It was also noted that low uptake among children in the present study was in contrary to widely advocated goal that high coverage should be attained in children less than 15 years [16, 17]. This is due to the fact that the average duration of trachoma infection at younger ages is long since tears and secretions infected with chlamydia are easily and frequently swapped among the young preschool children and their caretakers, which leads to repeated episodes of reinfection [27, 28].

Out of the total 1227 household heads who had ever taken the drug, 538 (43.9%) of them reported to have experienced side effects of azithromycin. Among these, 83 (15.4%) of them claimed that they had serious adverse effect of the drug. Another study conducted in Ethiopia reported that the prevalence of adverse events ranged 4.9**–**7.0% in children of 1–9 years of age and 17.0**–**18.7% in persons ≥10 years of age [29]. As it is pointed out in a recent study, patients taking azithromycin had an increased risk of cardiovascular death as compared to those who didn’t take [30, 31]. Another very recent meta-analysis of observational studies of 5 cohorts evidenced that azithromycin use was not associated with higher risk of death particularly in younger population whereas older population might be at higher risk of death (HR = 1.64 (CI, 1.232.19), *I* = 4%) [32]. Giving careful consideration on the safety impact of the mass treatment in special population such as elderly and patients with co-morbidity is mandatory [33].

Prior awareness of household heads’ about AMT has a positive association with the drug uptake (AOR = 7.19; 95% CI [3.27–15.82]). Household heads who thought that the mass treatment is beneficial were more likely to take the treatment as compared to those who believed the opposite (AOR = 7.33; 95% CI [4.13–13.02]). Another study documented that increased knowledge about the drugs given in mass treatment and their side effects may result in a better perception of its benefits than its barriers [34]. Hence adequate health education as well as awareness creation programs should precede the mass treatment campaigns for better coverage and acceptability of the treatment program.

### Limitations of the study

The study depended on the report of household heads on the participation of himself or herself and other eligible household members in the six rounds of AMT campaigns. The accuracy of the response therefore depends on the ability of the respondents to recall. Hence our study was liable to recall bias. In addition the focus group discussion we had before the survey showed that there was a great public concern on the adverse effect of azithromycin in the first round of the campaigns. This public concern could have affected how respondents answered the survey, especially with regards to adverse events.

## Conclusion

The overall average self-reported coverage of AMT in all six campaigns was low. There is low coverage and acceptability of the treatment in the urban community as compared to the rural residents. Being female and urban resident and low awareness about trachoma and azithromycin have negatively affected the acceptability of the AMT by household heads and hence the coverage. Awareness creation and health education programs about trachoma and AMT are needed for effective implementation of trachoma elimination programs. Further work on why female household heads are associated with higher risk of non-participation in AMT is also warranted.

## Additional file

Additional file 1: Questionnaires used in the survey. (PDF 37 kb)

## Abbreviations

AMT: Azithromycin mass treatment
ANRS: Amhara National Regional States
AOR: Adjusted odds ratio
COR: Crude odds ratio
TF: Trachomatous inflammation follicular
TT: Trachomatous trichiasis
