# Supplementary material for: Coverage of azithromycin mass treatment for trachoma elimination in Northwestern Ethiopia: a community based cross-sectional study
Source: BMC Ophthalmol. 2018 Aug 6;18:193. doi: 10.1186/s12886-018-0868-1 (PMC6091195; doi:10.1186/s12886-018-0868-1)
Supplement: Supplementary file 1 — Questionnaires used in the survey. (PDF 37 kb) [file 12886_2018_868_MOESM1_ESM.pdf]

Questionnaire

Addis Ababa University

School of Pharmacy

Department of Pharmaceutics and social pharmacy

Kebele code No _________ **_Gote_ code No________**

Verbal consent form before conducting interview

Greeting, my name is ______________________________. I am working with the research team of the Department of Pharmaceutics and Social Pharmacy, School of Pharmacy, Addis Ababa University. I would like to ask you a few questions concerning your perception and experience regarding to azithromycin mass treatment as well as your family's azithromycin mass treatment taking experience. The interview would take 20-30 minutes of your time. The purpose of this study is to assess the communities' acceptability to azithromycin mass treatment. This will be helpful for improving the azithromycin mass treatment program for the future. Your participation is voluntary. You can refuse to answer any questions if you are not interested. You can also withdraw from the study at any time, and there is no any problem happening to you in the future. All your responses will remain strictly confidential. No one will have the access to the information you will give for me. I will not ask your name and it will not appear on the interview guide. The information you will provide is analyzed in aggregate form with the other participants.

If you have/will have any questions or problems, you can contact

1. Zelalem Tilahun (Principal Investigator) : +251923109539
2. Dr. Teferi Gedif (the research project Advisor): +251911684854

Do you understand all the information I gave you? A. yes B. No

If yes, do I have your permission to continue? A. yes B. No

If Yes, Continue to the Next Page (encircle or write the response accordingly)

If No, Skip to the next Respondent

Date of Interview_________________

Time Started __________________

Time Finished __________________

Section I: Socio demographic information of the household heads			
**Qu.No**	**Questions**	**Response alternatives**	**Skip Pattern**
**1.**	Sex	1. Male<br>2. female	
**2.**	How old are You ?	Age in years___________	
**3.**	What is your Job?	1. Student<br>2. Farmer<br>3. Merchant<br>4.Government employee<br>5. House wife<br>6. Jobless<br>7. Others, specify____________	
**4.**	What is your Religion?	1.Orthodox<br>2. Muslim<br>3. Protestant<br>4. Others, specify ___________	
**5.**	Educational status	1. Unable to read and write<br>2. Able to read and write but no formal education<br>3. Grade 1-4<br>4. Grade 4-8<br>5. Grade 9-12<br>6. College and above	
**6.**	Income per month/ wealth<br>6.1.For urban community (income/ month)	1. < 500 birr<br>2. 500-1000 birr<br>3. 1000-1500 birr<br>4. 1500-2000 birr<br>5. >2000birr	
	6.2. For rural community (wealth)	1. less than 2 cows, 3 sheep, 2 horse, and 3 *kada* land<br>2. 2-5 cows, 3-8 sheep, 2-4 horse and 3-8 *kada* land<br>3. 6-10 cows, 9-12 sheep, 5-6 horse and 9-12 *kada* land<br>4. Greater than 10 cows, 12 sheep, 6 horse and 12 *kada* land	

**7.**	Marital status	1. Single<br>2. Married<br>3. Divorced<br>4. Widowed	
**Section II: Questions related to Azithromycin mass treatment for the household head**			
**8.**	Have you heard about trachoma?	1. yes<br>2. No	If No, skip to 15
**9.**	From where have you heard about trachoma?	1. Family<br>2. Neighbors<br>3. School<br>4. Health professionals<br>5. Mass Media (TV, Radio, Magazines)	
**10.**	What do you think is the cause of trachoma?	1. ____________________<br>2. ____________________<br>3. ____________________	
**11.**	How trachoma is transmitted from one person to the other?	____________________<br>____________________<br>____________________	
**12.**	Is trachoma a preventable disease?	1. Yes<br>2. No	If No, skip to 14
**13.**	How can we prevent trachoma?	____________________<br>____________________<br>____________________	
**14.**	How trachoma is treated?	____________________<br>____________________	
**15.**	Have you heard of azithromycin mass treatment?	1. Yes<br>2. No	If No, skip to 17
**16.**	From where have you heard about the azithromycin mass treatment?	1. Family<br>2. Neighbors<br>3. School<br>4. Health professionals<br>5. Community leaders<br>6. Mass Media (TV, Radio, Magazines	

**17.**	Was it given in your kebele?	1. Yes<br>2. No	
**18.**	Do you know why azithromycin treatment is given free of charge?	1. Yes<br>2. No	If No, skip to 20
**19.**	Why it is given free of charge?	______________________________<br>______________________________	
**20.**	Is the treatment given to those willing to take?	1. Yes<br>2. No	
**21.**	Have you or any member of your household taken azithromycin?	1. Yes<br>2. No	If Yes, skip to 23
**22.**	Why you didn't take azithromycin?	____________________________<br>____________________________<br>____________________________	
**23.**	How many times you had taken?	1. One<br>2. Two times<br>3. Three times<br>4. Four times<br>5. Five times<br>6. Six times	
**24.**	Had you yourself taken the treatment given in 2012 (2005 E.C)?	1. Yes<br>2. No	If Yes, skip to 26
**25.**	Why you hadn.t taken the treatment given in 2012 (2005 E.C)?	_____________________________<br>______________________________<br>_____________________________	
**26.**	Do you think it is beneficial?	1. Yes<br>2. No	If No, skip to 28
**27.**	What did you benefited from it?	1. Increase of my eye health<br>2. decrease in intestinal infection<br>3. Others, specify____________<br>_________________________	
**28.**	If it is not beneficial, why did you take it?	1. _________________________<br>2. __________________________<br>3. __________________________	
**29.**	Did you experience any side effects after taking the drug?	1. Yes<br>2. No	If No. skip to 32

**30.**	What type of side effects had occurred on you?	1. Diarrhea<br>2. Nausea<br>3. Vomiting<br>4. Stomach ache<br>5. Heart burn<br>6. Anal burning sensation<br>7. Others, specify___________	
**31.**	Have you ever encountered with serious adverse experience of the drug?	1. Yes<br>2. No	
**32.**	Have you ever heard about other persons who were experienced side effects?	1. Yes<br>2. No	
**33.**	What side effects of the drug did you heard in other persons?	1. Diarrhea<br>2. Nausea<br>3. Vomiting<br>4. Stomach ache<br>5. Heart burn<br>6. Anal burning sensation<br>7. Others, specify__________	
**34.**	Have you ever heard serious adverse experience of the drug in other persons?	1. Yes<br>2. No	
**35.**	What serious adverse experiences you had heard?	1. Death<br>2. Disability<br>3. Birth defect<br>4. In patient hospitalization	
**36.**	Do you know any person encountered with serious adverse experience of the drug?	1. Yes<br>2. No	
**37.**	Have you ever used the drug for other purposes?	1.Yes<br>2. No	If No, skip to 39
**38.**	For what purpose?	1. ________________________<br>2. ________________________<br>3. ________________________	
**39.**	Did the drug treatment providers ask you about your previous illness before giving the treatment?	1. Yes<br>2. No	
**40.**	Did the drug treatment providers have provided any information when they gave you the drug?	1. Yes<br>2. No	If No, skip to 42

**41.**	What did they say?	1. ____________<br>2. ____________<br>3. ____________	
**42.**	Are you willing to take the drug if the mass treatment continuous in your kebele?	1.Yes<br>2. No	
**43.**	If No for question 43, what is your reason?	____________<br>____________<br>____________	

Section III: Questions related to Azithromycin mass treatment for the household head about his family members.

A. Socio demographic informations about household members

	Questions	House Hold Members						
		Wife	01	02	03	04	05	06
**44.**	Sex							
**45.**	Age in years	____	____	____	____	____	____	____
**46.**	Religion							
**47.**	Educational status							
**48.**	Marital status							
**49.**	Job							

B. Questions related to azithromycin mass treatment

**50.**	How many times had she/ he taken the treatment?							
**51.**	Did she/ he take the last treatment given in 2012?							
**52.**	If she/he didn't take the last treatment, why?							
**53.**	Is there any person who illed his /she eye right now?							
**54.**	What side effects had occurred on her/him after taking the drug in the last treatment?							
**55.**	Had you ever advised her/him not to take the drug?							
**56.**	For what other purpose did your families use the drug?							

Thank you for your cooperation
